# AC-UNet: an improved UNet-based method for stem and leaf segmentation in Betula luminifera

**DOI:** 10.3389/fpls.2023.1268098

**Published:** 2023-11-27

**Authors:** Xiaomei Yi, Jiaoping Wang, Peng Wu, Guoying Wang, Lufeng Mo, Xiongwei Lou, Hao Liang, Huahong Huang, Erpei Lin, Brian Tapiwanashe Maponde, Chaihui Lv

**Affiliations:** ^1^ College of Mathematics and Computer Science, Zhejiang A&F University, Hangzhou, China; ^2^ Hangzhou Ganzhi Technology Co Ltd., Hangzhou, China

**Keywords:** Betula luminifera, stem and leaf division, UNET, hollow space pyramidal pooling, crossed attention

## Abstract

Plant phenotypic traits play an important role in understanding plant growth dynamics and complex genetic traits. In phenotyping, the segmentation of plant organs, such as leaves and stems, helps in automatically monitoring growth and improving screening efficiency for large-scale genetic breeding. In this paper, we propose an AC-UNet stem and leaf segmentation algorithm based on an improved UNet. This algorithm aims to address the issues of feature edge information loss and sample breakage in the segmentation of plant organs, specifically in Betula luminifera. The method replaces the backbone feature extraction network of UNet with VGG16 to reduce the redundancy of network information. It adds a multi-scale mechanism in the splicing part, an optimized hollow space pyramid pooling module, and a cross-attention mechanism in the expanding network part at the output end to obtain deeper feature information. Additionally, Dice_Boundary is introduced as a loss function in the back-end of the algorithm to circumvent the sample distribution imbalance problem. The PSPNet model achieves mIoU of 58.76%, mPA of 73.24%, and Precision of 66.90%, the DeepLabV3 model achieves mIoU of 82.13%, mPA of 91.47%, and Precision of 87.73%, on the data set. The traditional UNet model achieves mIoU of 84.45%, mPA of 91.11%, and Precision of 90.63%, and the Swin-UNet model achieves . The mIoU is 79.02%, mPA is 85.99%, and Precision is 88.73%. The AC-UNet proposed in this article achieved excellent performance on the Swin-UNet dataset, with mIoU, mPA, and Precision of 87.50%, 92.71%, and 93.69% respectively, which are better than the selected PSPNet, DeepLabV3, traditional UNet, and Swin-UNet. Commonly used semantic segmentation algorithms. Experiments show that the algorithm in this paper can not only achieve efficient segmentation of the stem and leaves of Betula luminifera but also outperforms the existing state-of-the-art algorithms in terms of both speed. This can provide more accurate auxiliary support for the subsequent acquisition of plant phenotypic traits.

## 1 Introduction

Plant phenotyping is an emerging science that links genetics with plant physiology, ecology, and agriculture (Li et al., 2020). Plant phenotypes, at the latent level, allow for the extraction of important traits such as plant size, shape, and growth dynamics (Zhou et al., 2018). At a deeper level, they can reflect physical, physiological, and biochemical traits that characterize the structure and function of plant cells, tissues, organs, plants, and populations. Plant stem and leaf segmentation are important for obtaining plant phenotypic traits at different growth cycles. The key to plant phenotypic analysis is the effective and correct segmentation of plant organs. Since 1990, research related to plant organ segmentation, especially for diseased leaf identification, has been emerging. The phenotyping of 2D images is usually based on traditional image processing, machine learning, and pattern recognition algorithms, such as threshold-based segmentation (Fu et al., 2019), edge detection (Wang et al., 2018b), region growing (Scharr et al., 2016), clustering (Abinaya and Roomi, 2016), and their combined extensions to each other (Kalyoncu and Toygar, 2015; Pape and Klukas, 2015a; Pape and Klukas, 2015b). Although the above methods can also achieve image classification and segmentation, the results are slightly less satisfactory. In recent years, with the application of deep learning in phenotypic data parsing, a new perspective has been taken to solve some of the data parsing bottlenecks encountered in phenomics research. In particular, deep learning has made a major breakthrough in the field of semantic segmentation. Deep learning, based on convolutional neural networks (CNN), has reached an advanced level in image classification and segmentation. Sadeghi-Tehran P et al. (Sadeghi-Tehran et al., 2019) segmented images into hyperparameters by using simple linear iterative clustering to obtain canopy-related features. These features were then fed into a CNN classification model to achieve semantic segmentation of wheat ears. Tamvakis P N et al. (Tamvakis et al., 2022) used deep learning methods (supervised and unsupervised learning-based approaches) to semantically segment images of grape leaves. They developed an automatic leaf phenotype analysis object detection system by segmentation that generates information about the structure and function of the leaves. Frank Gyan Okyere et al. (Okyere et al., 2023) developed a neural network-based segmentation tool to achieve high-throughput phenotypic analysis of cylinder beans and wheat. Conventional segmentation methods are more commonly implemented for the segmentation of crop stems, leaves, and fruits, and less frequently for forestry applications.

Betula luminifera is an economically valuable forest tree widely distributed in China. It is commonly found in the southern region of the Qinling Mountains and Huaihe River, at elevations ranging from 600 to 1700 meters above sea level. The wood of Betula luminifera is excellent, with a yellowish or reddish-brown color, fine texture, and hardness, making it highly valuable with diverse applications.To address the efficient acquisition of plant phenotypic traits, this study utilized self-collected images of Betula luminifera. It employed a modified version of the traditional UNet (Ronneberger et al., 2015), replacing the coding part with the VGG16 backbone feature extraction network (Simonyan and Zisserman, 2014; Deng et al., 2009). Additionally, the study introduced the ASPP module (Chen et al., 2018); (Chen et al., 2018); (Chen et al., 2017) with further improvements (He et al., 2016) (Wang et al., 2018a) and incorporated the cross-attention mechanism (CCA) (Huang et al., 2018). For the loss function, the compound loss Dice_Boundary (Kervadec et al., 2019); (Ma et al., 2021) was employed. The resulting model, called AC-UNet, aimed to re-fine the segmentation of Betula luminifera’s stems and leaves. The study conducted experiments using Betula luminifera seedlings from the Pingshan Experimental Base of Zhejiang Agriculture and Forestry University in northwestern Zhejiang Province as test subjects. The experimental results were compared with those of PSPNet (Zhao et al., 2017), DeepLabV3 (Chen et al., 2018), UNet, and Swin-UNet (Cao et al., 2021),The findings demonstrated that the proposed model outperformed other segmentation algorithms in terms of performance. Specifically, it exhibited more detailed feature extraction along the edges of the plant stems and leaves, leading to an overall better plant restoration.

The contribution of this paper consists of the following two main parts:

1. Constructing a Betula luminifera dataset with Betula luminifera seedlings as the experimental object, based on three lineages, namely Taihuyuan in Hangzhou, Napo in Guangxi, and Anhua in Hunan.

2. A semantic segmentation method is proposed based on an improved UNet piggy-backing on ASPP and CCA. The model uses VGG16 as the backbone network to extract deep semantic features, piggybacks on ASPP modules with appropriate hole expansion rates set, fuses the CCA mechanism with Dice_Boundary loss, and captures long-range global feature information by reducing the number of network parameters and network depth to obtain multiscale semantic information and improve segmentation accuracy.

The construction of the data set meets the data needs of the scientific research community and ecologists for Betula glabra research, and provides a powerful tool for ecosystem monitoring and plant genetics research. The improved UNet semantic segmentation technology comes from the challenges encountered in light bark research. With a deep understanding of the complexity of seedlings and the limitations of traditional segmentation methods, detailed experiments have demonstrated the significant performance of this algorithm in processing Betula glabra data sets. We believe that this method is not only applicable to Betula glabra but also has the potential to Its wide range of applications include medical image segmentation, cartography, and botanical research.

## 2 Materials and methods

### 2.1 Experimental dataset

A forestry dataset was constructed based on image segmentation of Betula luminifera seedlings to obtain a large amount of accurate data about Betula luminifera. These images are used to segment plant organs such as leaves and stems, providing an effective aid for plant monitoring and plant phenotype analysis.

Seedling cultivation and data collection were carried out at the Pingshan Experimental Base of Zhejiang Agriculture and Forestry University. The study area is located in Lin’an District, Hangzhou City, Zhejiang Province, at Zhejiang Agriculture and Forestry University, Jincheng Street, with geographical coordinates ranging from 118°51’ to 119°52’ E and 29°56’ to 30°23’ N. The area has a subtropical monsoon climate with four distinct seasons, abundant light, and rainfall, making it suitable for the cultivation of Betula luminifera seedlings. The three seedlings selected for this study were sourced from provinces south of their natural distribution in the Qinling and Huaihe River basins (Zhejiang, Guangxi, and Hunan) and covered a wide range of seedling morphology to verify the applicability of the method to different types of plants. The seedlings for data collection were selected from the uniformly cultivated Betula luminifera at the Pingshan Experimental Base of Zhejiang Agriculture and Forestry University. There were a total of 300 plants. The cultivation took about 30 days. The height of the plants ranged from 10 to 35 cm, and the seedlings grew upright. To facilitate data collection, each seedling was individually transplanted into a uniform calibre plastic pot for numbering. The cultivation greenhouse was maintained at a temperature of 23°C during the day with natural light, 20°C at night, and a uniform humidity setting of 70%.

**Figure 1:**
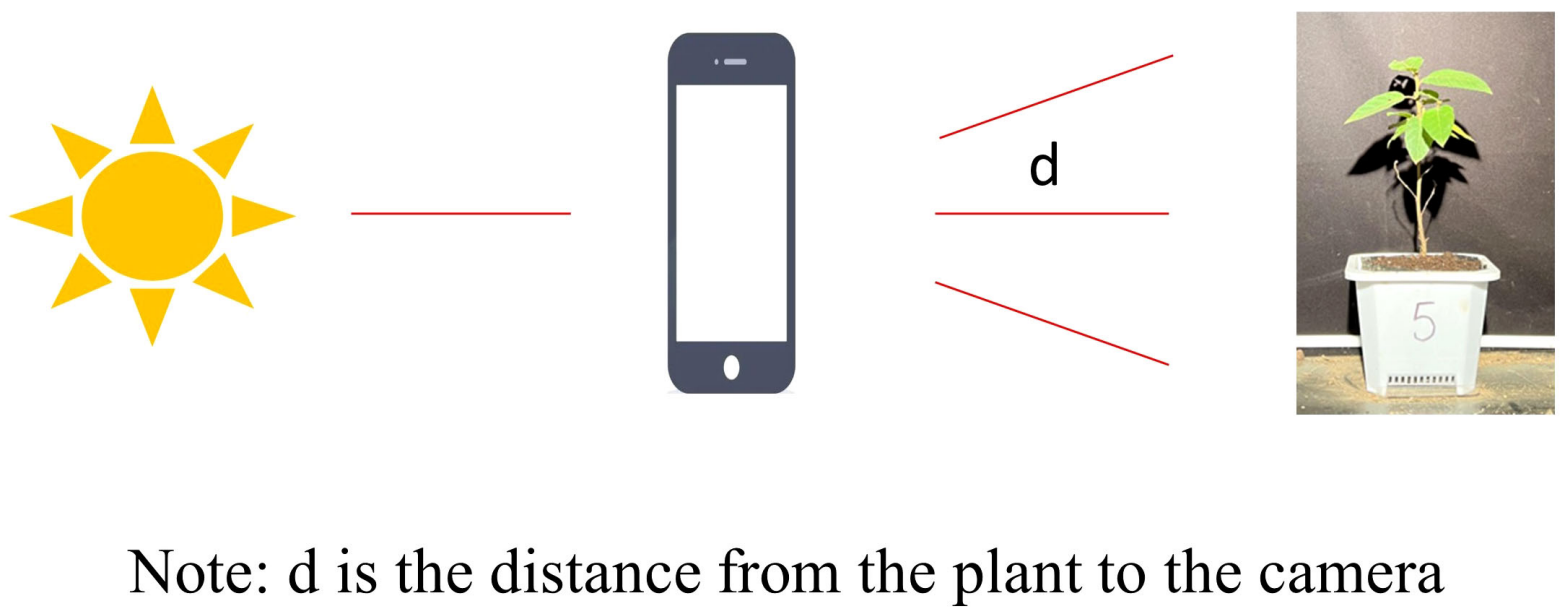
Diagram of the plant shot.

In this study, black velvet cloth was used as the shooting background during collection to reduce the impact of background objects and light source scattering and provide a stable environment for plant shooting. The iPhone 13 Pro Max mobile phone is used as a plant shooting device to obtain high-quality plant images. The same shooting device is always used during collection, which stabilizes the image quality and helps capture the microscopic details of the seedlings. Image collection will be carried out at different times in November 2022 and December 2022 to ensure that the collected data sets are in a stable growth state at the same stage. During filming, the lens was held flush with the target of the photographed plant, and the distance from the plant sample to the lens was kept at d = 90 cm (**Figure 1**). The plants were placed on a tray at the bottom of the platform, and they were rotated in turn at 90-degree clockwise angles. Multiple angles were taken for the front, back, left, and right sides of each plant, with the light source placed at the viewpoint directly opposite the sample, pointing towards the photographed sample. A total of 1200 valid images were taken during this data collection process. After screening and culling (removing images with high longitudinal overlap and sparse foliage), 490 images were obtained, forming the original image dataset.

**Figure 2:**
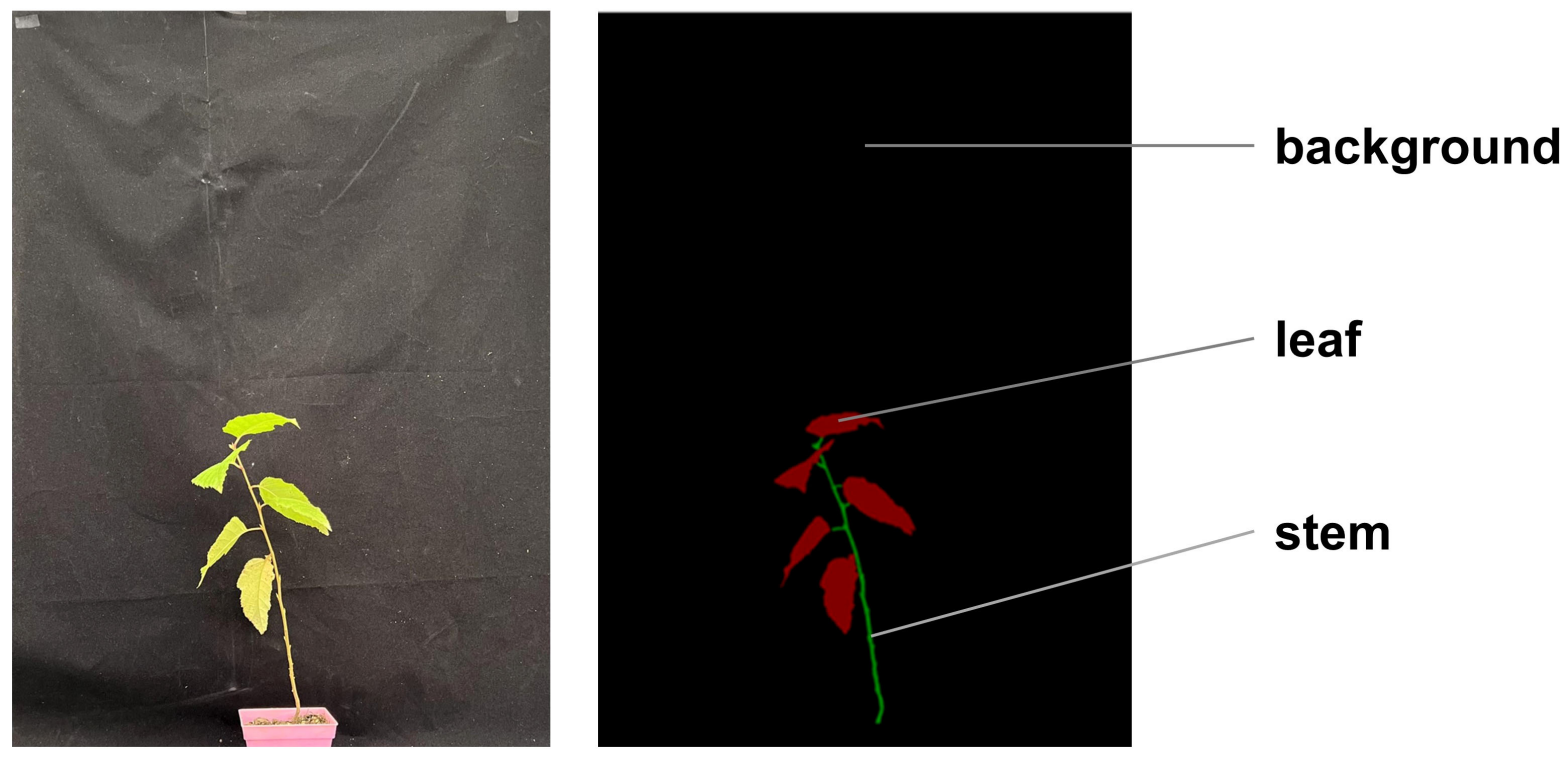
Schematic diagram of image annotation.

The plant stems and leaves in the original image dataset were accurately labeled using the Labelme image annotation software, as shown in **Figure 2**. The labeled images contain three semantic categories: the background part, stem, and leaf. The pixel values assigned to these categories are as follows: the background part is 0, the stem is 1, and the leaf is 2. The dataset consists of a total of 490 RGB labeled images, which were randomly divided into a training set and a validation set in a ratio of 9:1. This resulted in 442 training samples being inputted into the UNet model for training, while the remaining 48 im-ages were used for validation.

### 2.2 OTSU

The maximum variance between classes method, commonly known as the Otsu method, is a self-fitting method for automatically finding thresholds for the bimodal case. It was proposed by the Japanese scholar Nobuyuki Otsu in 1979 and is currently recognized as a relatively reasonable choice for threshold segmentation, yielding good segmentation results. As the name suggests, the method uses the idea of maximizing the variance between the target and background regions for segmenting images. In other words, the optimal threshold T is chosen to maximize the variance between the target and the background, defining the region smaller than the threshold T as D1 and the region larger than the threshold as D2. This allows the required region to be distinguished based on the threshold definition. The advantage of this method is its simplicity and speed of calculation. It is not easily affected by image brightness and contrast and is widely used in image binarization segmentation.

### 2.3 HSV colour threshold splitting

HSV is a color space based on the intuitive properties of color, created by A.R. Smith in 1978 and also known as the hexagonal cone model. It is a color system that is more commonly used in people’s lives compared to RGB. HSV is commonly found in TV remote controls, painting palettes, and brightness adjustments in video software. Subjectively, the HSV color system aligns more with how people describe color. The parameters of color in this system are: Hue, Saturation, and Value.

Hue (H) represents popular perceptions of colors like red, green, blue, etc. However, more refined expressions can be used such as plum red, magenta, grass green, dark green, and so on.

Saturation (S) refers to the intensity or shade of a color. It is a concept that takes values in the range of 0-100%. For example, in the case of red, bright red has high saturation as it represents a pure color. If mixed with other shades of color, the saturation decreases, such as in the case of pink.

Value (V) represents brightness or purity of color, ranging from 0 to 100%. This value is commonly used when adjusting the brightness of a screen.

Referring to the color range table in **Table 1**, the region of interest (ROI) in the image is selected. Generally, before carrying out this step, denoising is required. However, in this paper, since it is based on the mask map of the prediction results and there is no noise effect, denoising is performed directly on the extracted leaf part. 

**Table 1:** HSV colour space colour range.

	black	gray	white	red		orange	yellow	green	cyan	blue	purple
Hmin	0	0	0	0	156	11	26	35	78	100	125
Hmax	180	180	180	10	180	25	34	77	99	124	155
Smin	0	0	0	43		43	43	43	43	43	43
Smax	255	43	30	255		255	255	255	255	255	255
Vmin	0	46	221	46		46	46	46	46	46	46
Vmax	46	220	255	255		255	255	255	255	255	255

### 2.4 Improved UNet-based plant image segmentation algorithm

#### 2.4.1 Network architecture

**Figure 3:**
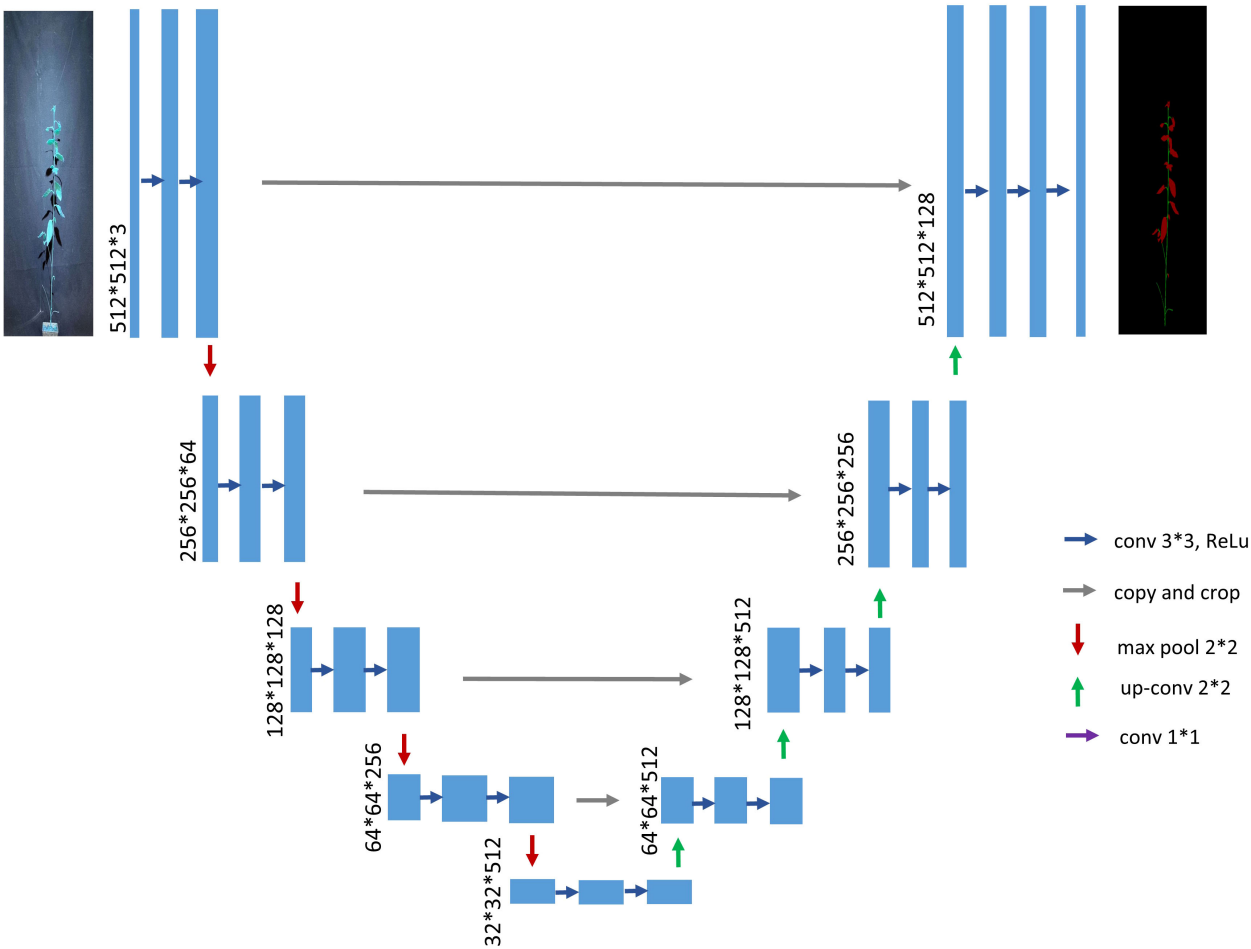
Traditional UNet network architecture diagram.

The UNet network (Ronneberger et al., 2015) was proposed in 2015, and at the time of its mention, its main application was semantic segmentation of medical images. The emergence of UNet has greatly reduced the amount of data required for training deep learning neural networks, which originally required thousands of annotated data to be trained, and it pioneered the application of neural networks to image segmentation. This network is still widely used despite the birth of many segmentation networks. The structure of the traditional UNet model is shown in **Figure 3**. The highlight of this structure is that the whole network presents a U-shaped structure, hence the name UNet. The UNet network is very simple, with the first half acting as feature extraction and the second half as upsampling. This structure is also called an encoder-decoder structure in some literature. The downsampling part refers to the basic structure of a convolutional neural network, with two convolutional units composed of 3x3 convolutions, each followed by a ReLU and a 2x2 maximum pooling, while doubling the number of feature channels to capture context for feature extraction and learning. In the upsampling section, two convolutional units composed of 3x3 convolutions, immediately followed by 2x2 convolutions, halve the number of feature channels that were originally doubled and concatenate them with the corresponding cropped feature maps in the encoding section. The missing values at the boundaries after each previous convolution step are filled in. Finally, each 64-component feature vector is mapped to the desired number of classes using a 1x1 convolutional unit.

The UNet model achieves superior segmentation results on various datasets. The feature information of plants itself is relatively stable, and there are no special or novel feature information. Therefore, both high-level semantic features and low-level semantic features are extremely important. As one of the current excellent semantic segmentation networks, the UNet network also has some shortcomings. Firstly, as each pixel point needs to take a patch, it makes the patches of two neighboring pixel points too similar, resulting in a significant amount of redundancy. This redundancy not only leads to a poor segmentation situation but also reduces the training speed of the network. Secondly, it is challenging to achieve both localization accuracy and access to contextual information simultaneously. The larger the patch size, the more maximum pooling layers are required, which in turn reduces the localization accuracy. Additionally, as the number of pooling layers increases, more information is lost.

**Figure 4:**
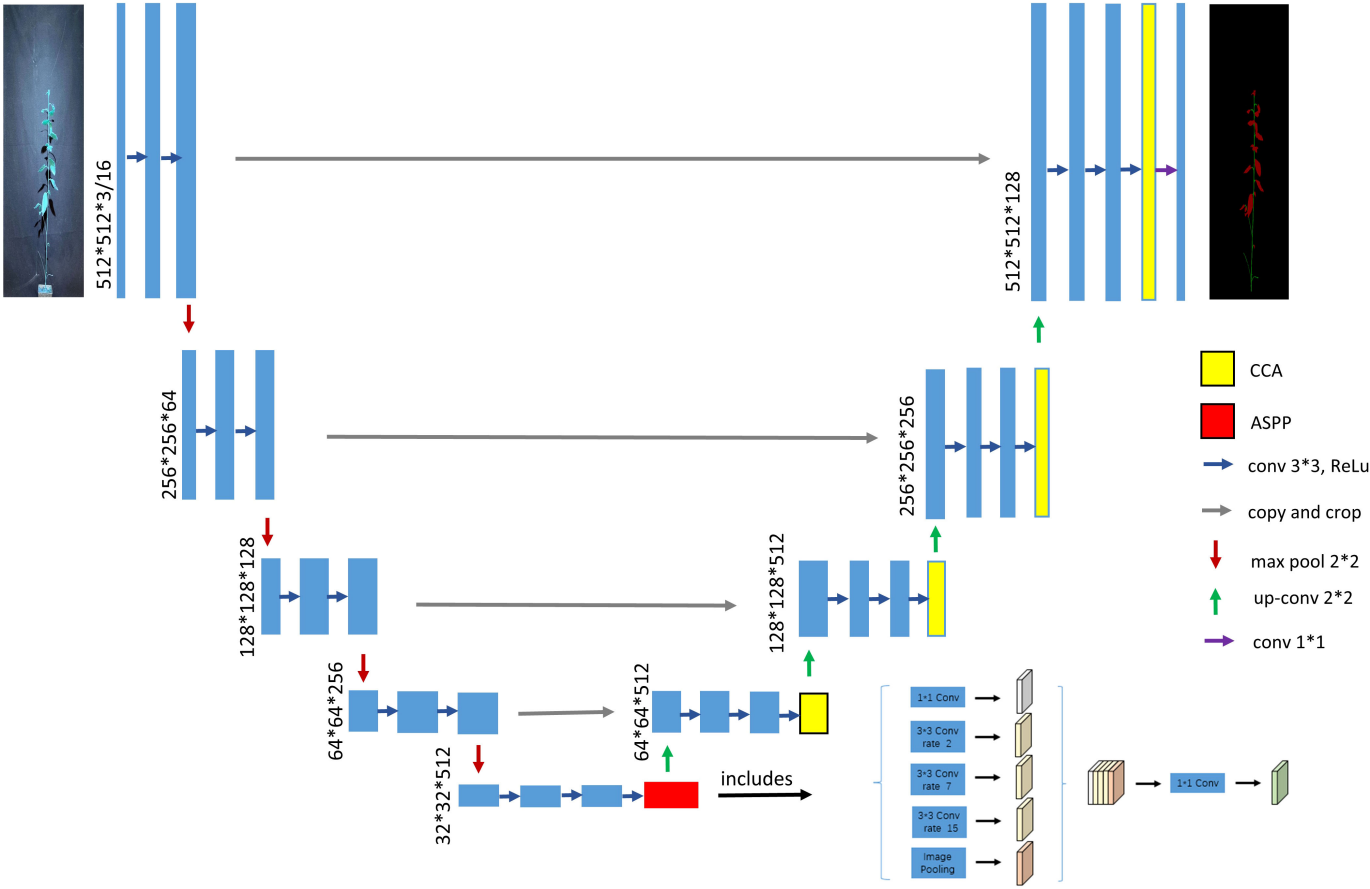
Diagram of the improved UNet network architecture.

Then, directly inputting the shallow network information into the decoder part will cause a low rate of obtaining semantic information of stem-and-leaf edges, resulting in poor segmentation accuracy. To improve the training performance and address the aforementioned deficiencies, this paper introduces the following improvements to the traditional UNet model architecture:

(1) Using VGG16 as the main stem feature extraction part of UNet (Ronneberger et al., 2015), which significantly reduces the amount of parameter computation of the model, decreases memory occupation, and improves the computational speed.

(2) Introducing an improved ASPP module (Chen et al., 2018) in the middle of the encoder and de-coder, which expands the sensory field without losing semantic information and enhances the network’s feature extraction capability.

(3) Introducing CCA (Huang et al., 2018) in the decoding part to reduce GPU memory usage and im-prove model segmentation accuracy.

(4) Replacing the loss function with Dice_Boundary composite loss (Kervadec et al., 2019); (Ma et al., 2021) to ad-dress the imbalance of pixel distribution between categories. The improved model structure is shown in **Figure 4**.

#### 2.4.2 Optimised feature extraction module

**Figure 5:**
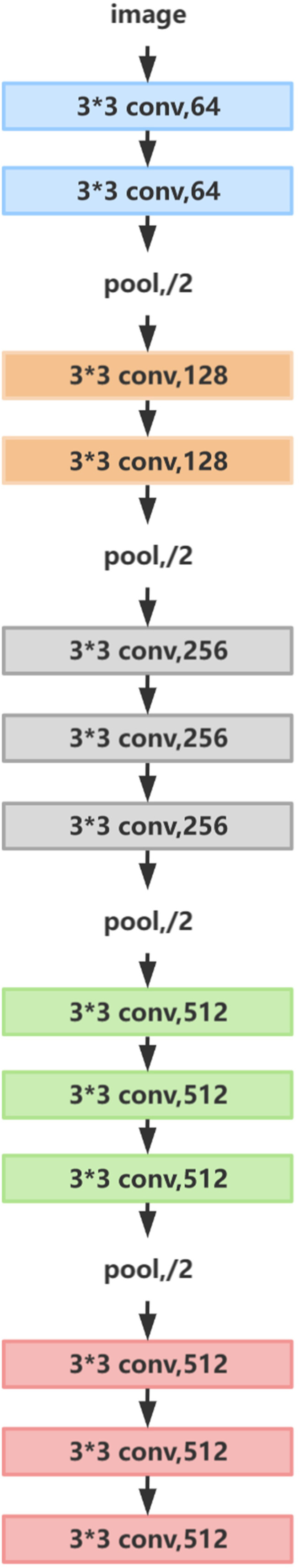
Optimised backbone feature extraction module.

The external environment presents various interferences in the Betula luminifera im-age acquisition process. Additionally, the UNet network itself employs a specific number of convolution kernels in the encoding process to extract image features. This multi-step convolutional operation leads to excessive redundancy in the feature map of the segmentation model, resulting in poor semantic interpretation of Betula luminifera images and reduced network training speed. To reduce parameter redundancy within the UNet network, enhance network depth for improved classification accuracy, and extract more abstract higher-order features from the image, VGG16 is utilized as the backbone feature network of UNet. The use of pre-trained mature models significantly enhances the training speed of the UNet network while ensuring accuracy. Compared with other networks, VGG16 can employ multiple 3x3 convolutional kernels instead of large-scale convolutional kernels, thus reducing the number of parameters during network operation. In this algorithm, the three fully connected layers of VGG16 are omitted due to their excessive consumption of computational resources. Moreover, VGG16 and VGG19 have demonstrated better segmentation effects in practical applications. Compared with VGG19, VGG16 has three fewer convolutional layers, making it a shallower network. Given that the network’s quality is ensured, this paper selects the network with fewer parameters. The structure of VGG16 as the main feature extraction network is depicted in **Figure 5**. Furthermore, this paper adopts pre-training weights from Imagenet (Deng et al., 2009) for transfer learning to improve the model’s generalization.

#### 2.4.3 Fused multidimensional feature acquisition

Betula luminifera images contain multi-scale objects, such as small leaves versus larger leaves, smooth branches versus branches with small forks, and so on. The image is subjected to continuous convolution and pooling or other downsampling operations in the network to integrate the multi-scale contextual information, which tends to result in low-resolution feature maps in the network, making it impossible to reconstruct the image details.In order to overcome the disadvantages of local information loss and lack of correlation of distant information due to the grid effect when using a single null convolution, the expansion rate is changed based on the original null convolution in the ASPP module (Chen et al., 2018). A type of null convolution that can increase the sensory field while still maintaining sensitivity to details is proposed. The optimized cavity convolution can effectively expand the receptive field of the convolution kernel to incorporate larger contextual information without increasing the number of parameters and computational effort.

**Figure 6:**
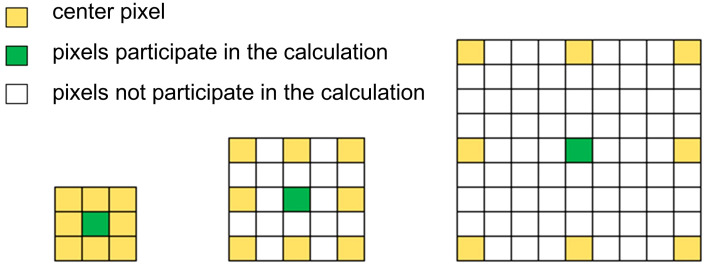
Field of perception for convolution at different expansion rates R.

In the semantic segmentation algorithm, the two-dimensional hole convolution is achieved by inserting 0 between each pixel of the convolution kernel. For a convolution kernel with a size of k×k, the size after the hole convolution is kd×kd, where kd = k + (k‒1) × (r‒1). **Figure 6** shows the size of the convolution kernel receptive fields at different expansion rates, and the dilated convolution receptive fields with expansion rates of 1, 2, and 4 are 3×3, 5×5, and 9×9, respectively.

**Figure 7:**
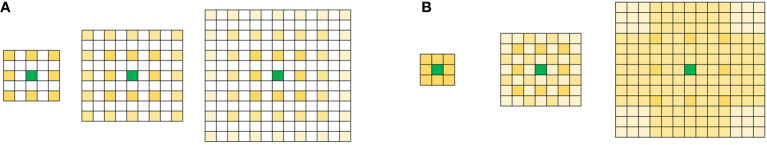
Null convolution **(A)** Null convolution “lattice effect”.**(B)** Combined hole convolution with reasonable expansion rate.

The ASPP module uses multiple parallel cavity convolution layers with different sampling rates. The features extracted for each sampling rate are further processed in separate branches and fused to generate the final result. This approach allows for the extraction of multiscale features of the object and image context while ensuring high image resolution. Wang P (Wang et al., 2018a) et al. found that improper settings of the original parallel cavity convolution expansion rate could easily cause a “grid effect,” as shown in **Figure 7A**.

A reasonable expansion rate setting should be as shown in **Figure 7B**, which not only avoids the loss of relevant information but also captures the target context at different scales. Let’s define the maximum distance between the nonzero values of the convolution kernels of the ith layer as follows:


(1) $$M_{i}=max\left[M_{i+1}-2r_{i},M_{i+1}-2\left(M_{i+1}-r_{i}\right),r_{i}\right]\left(M_{n}=r_{n}\right)$$

According to the literature (Wang et al., 2018a), the void convolution’s growth rate should adhere to the following theory: If the expansion rates for N convolutions and void convolutions of size K*K are figured to be [r1, r2,…, ri,…, rn], then the formula satisfies M_2 k, where ri de-notes the ith void convolution’s expansion rate and M_i denotes the ith layer’s maximum expansion rate. There shouldn’t be a common factor relationship among the growth rates of the same group (e.g., 2, 4, 8, etc.), as this will still result in lattice effects. For example, when K = 3 and r = [1, 2, 5], no lattice effect will occur after validation; however, when K = 3 and r = [1, 2, 9], which do not meet the requirements after validation, a lattice effect will occur.

**Figure 8:**
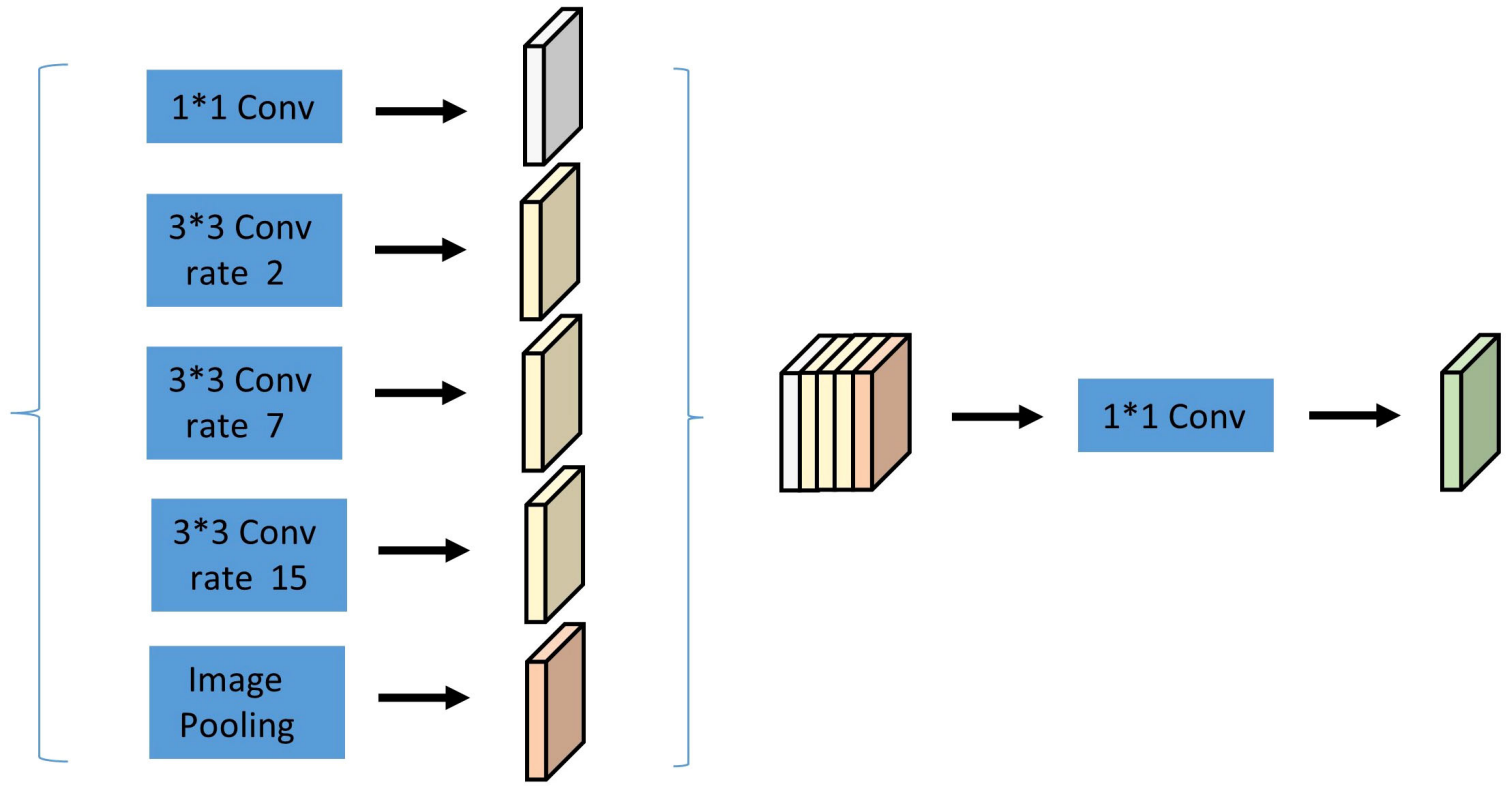
Hollow Pyramid Pooling Module.

Therefore, this paper follows the above design principles and uses multiple parallel convolution layers with different sampling rates. Experimentally, it resets a set of null convolutions with expansion rates of 1, 2, 7, and 15 (M_i = 3 = k). The features extracted from each sampling rate are processed separately and fused into the final result. The adjusted ASPP module is shown in **Figure 8**.

As shown above, after the fourth block, the parallel architecture is added. The left side of the parallel part consists of a 1x1 convolution, three 3x3 convolution kernels (with void rates of 2, 7, and 15), and pooling operations. The right side represents the image-level features, wherein the features are globally pooled on average, convolved, and then fused. After parallelization and subsequent convolution with 256 1x1 convolution kernels (the convolution layer is followed by a BN layer), the resulting feature map is upsampled to the desired feature dimension.

#### 2.4.4 Integrating efficient attention

The influence of environmental factors such as temperature, humidity, and light intensity leads to variations in the growth states of each Betula luminifera plant. Consequently, there are difficulties in acquiring features. The attention mechanism, inspired by the human attention mechanism, focuses on important information features. The objective of this study is to achieve high-precision segmentation of Betula luminifera stems and leaves. By focusing on different feature pheromones of stems and leaves to achieve a higher precision segmentation effect, cross attention (CCA) is introduced to enable the network to learn more interested regions, thus avoiding the loss of too much semantic information. This enhancement leads to improved segmentation performance of the model.

The Non-local approach is proposed to address the problem of long dependencies (Wang et al, 2021). In this case, the CCA module (Huang et al., 2018) replaces the global attention mechanism in Non-local with a cross-shaped attention mechanism. This modification allows individual pixels to obtain global contextual dependencies through the cross module, thanks to a double-loop operation. As a result, it effectively enhances feature extraction and achieves leading performance in segmentation-based benchmarks. Moreover, the CCA module is GPU memory friendly, providing a significant solution to the issue of excessive parameters in the UNet network and the resource consumption during the model training process.

Compared to Non-local, CCA reduces the FLOPS by 85%. The input image undergoes feature extraction by the backbone network, and the fused CCA not only mitigates the loss of local information but also captures long-distance global information, thereby improving the network’s feature extraction capability.

**Figure 9:**
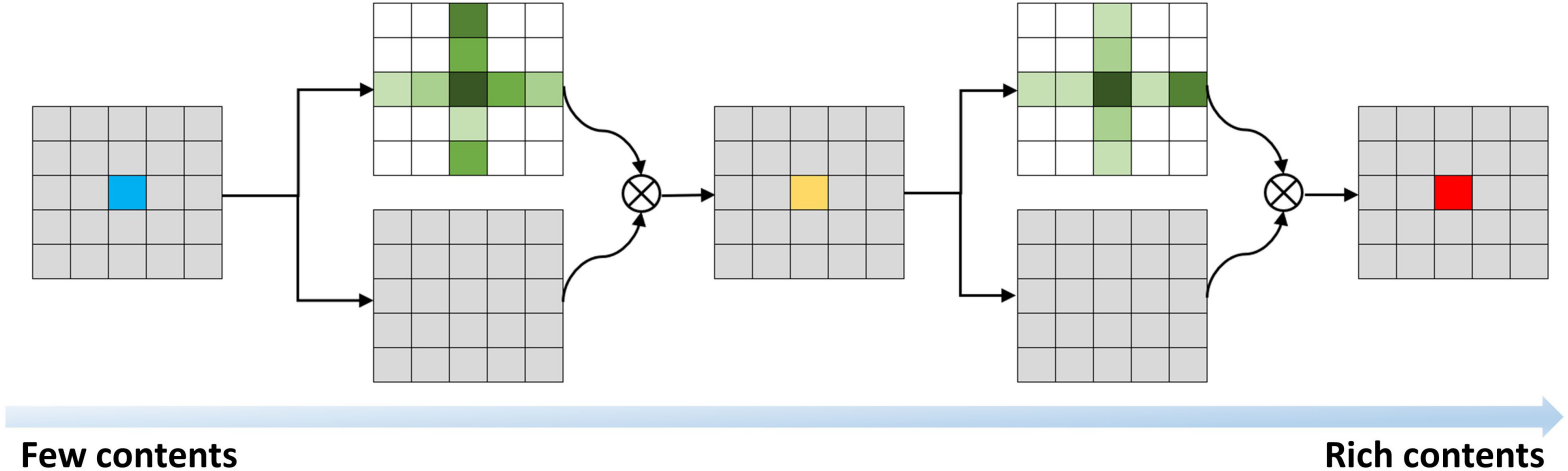
CCA module.

In summary, this paper introduces the CCA module in the decoding part, which operates through the attention mechanism illustrated in **Figure 9**.

#### 2.4.5 Optimisation of loss functions

Unlike conventional semantic segmentation of objects, the stem and leaf segmentation of Betula luminifera has fewer categories, which include three parts: stem, leaf, and background. The other two categories are more challenging to distinguish overall due to the uncertainty in the proportion of different plant length distributions. They are also several orders of magnitude smaller than the spatial occupation of the background. There is a well-known drawback to the highly unbalanced problem in that it assumes all samples and categories are of equal importance. This assumption typically leads to instability in training and results in decision boundaries that are biased towards the more numerous categories.

To address the above imbalance problem and consider the image as a whole, this paper combines Dice loss (Ma et al., 2021) with Boundary loss (Kervadec et al., 2019) and introduces a compound loss function, Dice_Boundary, for optimizing the loss of the training model. The function is defined as follows:


(2) $$l_{DiceBoundary}=l_{Dice}+l_{Boundary}$$

An ensemble similarity measure function called the former Dice loss function is utilised to lessen the inaccuracy between the segmented and labelled images. It is typically used to determine how similar two samples are, producing results in the [0,1] range. Greater overlap between the expected and actual results, which denotes improved performance, is shown by a larger score. In contrast, a lower loss value is preferred, as shown in equation (3).


(3) $$l_{\mathrm{Dice}}=1-\frac{2\sum_{c=1}^{C}\sum_{i=1}^{N}g_{i}^{c}s_{i}^{c}}{\sum_{c=1}^{C}\sum_{i=1}^{N}g_{i}^{c}+\sum_{c=1}^{C}\sum_{i=1}^{N}s_{i}^{c}}$$

where denotes the predicted label, denotes the true label, N is the number of pixels in the image, and C is the number of classifications. The latter Boundary loss function takes the form of a contour space rather than a region metric to alleviate the difficulties of the highly unbalanced problem. In addition, the Boundary loss supplements the region in-formation and is usually applied to segmentation tasks with a high imbalance, as shown in equation (4).


(4) $$Dist\left(\partial G,\partial S\right)=\int_{\partial G}{\left\|y_{\partial S}\left(p\right)-p\right\|}^{2}dp$$

where ‘p’ denotes a point on the edge of ‘A’, and ‘q’ is the corresponding point on ‘B’, in other words, ‘q’ represents the intersection with ‘A’ found at point ‘p’ on ‘B’. ‘||.||’ de-notes the paradigm.


(5) $$\mathrm{Dist}\left(\partial G,\partial S\right)\approx 2\int_{\Delta S}D_{G}\left(q\right)\mathrm{dq}$$

where ΔS denotes the distance between even contours, and D is the distance map relative to the boundary, in other words, D denotes the distance between any point q and the nearest point on the contour. Equation (5) is used to derive equation (6).


(6) $$\mathrm{Dist}\left(\partial G,\partial S\right)=2\left(c\left(q\right)s\left(q\right)\mathrm{dq}-\int_{\Omega }\emptyset_{G}\left(q\right)g\left(q\right)\mathrm{dq}\right)$$

(7) $$l_{\mathrm{Boundary}}=\int_{\Omega }\emptyset_{G}\left(q\right)S_{\theta }\left(q\right)\mathrm{dq}$$

where S is the binary indicator function of the region S and is the horizontal set representation of the boundary. For S = [value], the softmax output of the network replaces [value] in equation (7) to obtain the boundary loss of equation (6).

## 3 Experimental analysis and discussion

### 3.1 Data preprocessing

#### 3.1.1 Shearing

Image clipping is an operation that cuts out a specific area in an image. Clipping can remove noise and avoid large-area image exposure problems; highlight areas of interest, reduce interference, and make the image more focused on the target area; resize, adjust For a specific input size, it facilitates the processing of the model. The images collected in this research have problems such as overexposure of light, unprotrusive regions of interest, images taken by mobile phones, and inconsistent image sizes. Therefore, the cutting method is used to preprocess the images to meet the needs of the algorithm and improve the efficiency of image processing. quality and effect.

#### 3.1.2 Median filtering

Images are subject to temperature and humidity, magnetic fields, losses during signal transmission, vibration noise, etc., during formation or transmission, resulting in degradation of image quality and distortion of the final imaging results. These factors inevitably have an impact on later image analysis and research. In the image acquisition of this study, the irradiation of the physical light source and natural light at different times of the day caused greater disturbance to the subsequent image processing work. To reduce the effects of noise, noise reduction was applied using image filtering processing.

Based on the characteristics of the collected Betula luminifera images, a spatial domain filtering process is used. Spatial domain filtering consists of linear and non-linear filtering. In this study, the final approach utilizes the median filtering algorithm in non-linear filtering to achieve noise reduction processing of the images. Median filtering has the effect of removing impulse noise and preserving edge details of the image.

As a typical type of non-linear smoothing filter, the basic principle of median filtering is to replace the value of a point in a digital image or digital sequence with the median of the values of the points in a neighborhood of that point. This allows pixels with a relatively large difference in gray value compared to the surrounding pixels to be replaced, effectively eliminating isolated noise points. The median filtering formula can be expressed as:


(8) $$F_{i}=Med\left\{F_{i-m},\ldots ,F_{i},\ldots ,F_{i+m}\right\}$$

(For a one-dimensional sequence, taking p numbers for median processing, m = (p-1)/2.)

For two-dimensional images undergoing median filtering, the filter window is also two-dimensional. This window can take on various shapes, including lines, squares, circles, crosses, and so on. The formula for two-dimensional median filtering can be expressed as:


(9) $$F_{i,j}=Med\left\{f_{i,j}\right\}$$

(Med for the number of filter windows)

**Figure 10:**
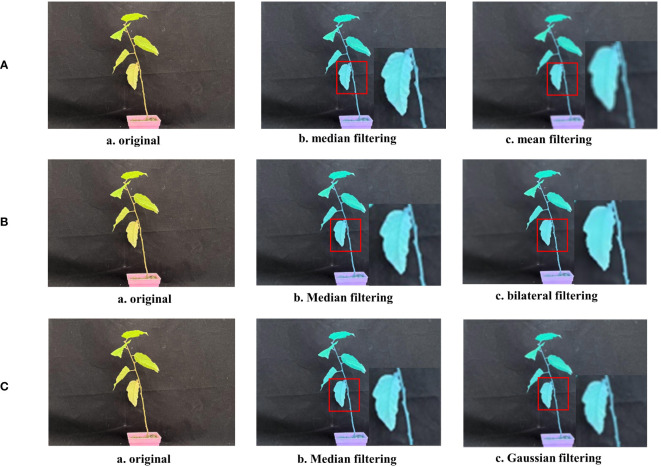
Comparison of filtering methods. **(A)** Median filtering VS Mean filtering. **(B)** Median filtering VS Bilateral filtering. **(C)** Median filtering VS Gaussian filtering.

Mean filtering is a common linear filtering algorithm that determines the average of the noise components, as the name suggests. In the procedure, the average value of the adjacent pixels in a template is used to replace the original pixel value. The target pixel itself and the eight pixels around it that are centred on it make up the template. Unlike mean filtering, this method preserves the image’s edge information for further image processing while also addressing the problems of blurring image details and loss of features (**Figure 10A**).

Bilateral filtering, like median filtering, is a non-linear filtering method that combines the spatial proximity of an image and the similarity of pixel values in a compromise process. It takes into account both spatial domain information and grayscale similarity to achieve edge-preserving denoising. In this study, the output of the median filter is almost identical to the original image. The bilateral filter has little effect on the pixel values of the edges. However, due to the preservation of too much high-frequency information, the noise in the color image is not cleanly filtered, resulting in a lack of detailed texture, as seen in the figure with the missing leaf veins (**Figure 10B**).

Gaussian filtering is a linear smoothing filter that is suitable for removing Gaussian noise and is widely used in the noise reduction process of image processing. In layman’s terms, Gaussian filtering is the process of weighted averaging of the entire image. Each pixel’s value is obtained through a weighted average of its own value and the values of other pixels in its neighborhood. While the difference between the Gaussian filtered image and the median filtered image may not appear significant, the image processing speed is much slower with Gaussian filtering compared to median filtering (**Figure 10C**).

### 3.2 Experimental environment parameter settings and evaluation indicators

The experimental device has an NVIDIA GeForce RTX 3060 GPU, a 12th Gen Intel(R) Core(TM) i5-12400 2.50 GHz processor, and 12 GB of video memory. Windows 64-bit with CUDA 11.2 is the operating system used in the software environment. The deep learning framework is PyTorch, and Python is the programming language. As the deep learning framework, PyTorch was employed. A batch training approach was used to train the network. For training, different batches of the training and validation sets were created. the network model’s traversal of every image in the training set is calculated as one iteration. The network model is initialized using pre-trained weights to initialize the backbone network. The initial learning rate is 0.001 and is optimized by the Adam algorithm to calculate the adaptive learning rate for each weight parameter.

In order to quantify the segmentation effect of the segmentation method in this article on light bark images and compare the segmentation performance of different methods, the evaluation criteria introduced in this article mainly include category average pixel accuracy (mPA), precision (Precision), average intersection over union (mIoU) and F1 score. Among them, FN means that the model incorrectly classified it as a negative example, but it is actually a positive example; FP means that the model incorrectly classified it as a positive example, but it is actually a negative example; TP means that the model correctly classified it as a positive example, but it is actually a positive example; TN means The model correctly classified them as negative examples, but they were actually negative examples. The detailed division is shown in **Table 2**.

**Table 2:** Confusion matrix for classification results.

The Real Deal	Predicted results	
	Positive examples	Counter examples
Positive examples	True example	False counterexample
Counter examples	False example	true counterexample)

#### 3.2.1 Mean Pixel Accuracy

Mean pixel accuracy(mPA), Calculate the proportion of pixels per class that are correctly classified. mPA is expressed as:


(10) $$\text{Pi}=\frac{\text{TP}}{\text{TP}+\text{TF}}$$

(Note: Pi indicates pixel accuracy for each category)


(11) $$mPA=\frac{sum\left(Pi\right)}{class\;number}$$

#### 3.2.2 Precision

Precision, also known as the accuracy rate, measures the proportion of correct predictions (true cases) out of all predictions that are positive:


(12) $$Precision=\frac{TP}{TP+FP}$$

#### 3.2.3 Mean intersection over union

Mean Intersection over Union (mIoU) is the ratio of the intersection between the true label value and the predicted value to the union between the true value and the predicted value. mIoU is expressed as follows:


(13) $$mIoU=\frac{TP}{FP+FN+TP}$$

#### 3.2.4 F_1 Score

The F1-Score, also known as the Balanced F1-Score, is defined as the average sum of the precision and recall rates. The expression for the F-Score is:


(14) $$F_{1}=2*\frac{Precision*Recall}{Precision+Recall}$$

where Recall is expressed as the recall rate, which measures the probability of a category being correctly predicted among the true values, and the expression for Recall is:


(15) $$\text{Recall}=\frac{\text{TP}}{\text{TP}+\text{FN}}$$

FLOPs/G denotes the model complexity of the network and Params/M denotes the total number of parameters of the entire network model.

### 3.3 Backbone network performance comparison

In response to the problem of poor segmentation performance of stem and leaf details and edge parts in the experiment of Betula luminifera stem and leaf segmentation, two schemes are proposed to modify the UNet backbone network using the deep feature extraction network VGG16 and the feature extraction network ResNet50 with a residual network structure. Two types of UNet networks are trained under the same conditions to segment the stems and leaves of Betula luminifera, respectively. By comparing the effects of the two backbone networks on the UNet algorithm in stem and leaf segmentation, it is shown that UNet equipped with the VGG16 backbone network has a more precise detection effect, which is closer to the accuracy of the segmentation algorithm proposed in this article. Please refer to UNet-3 in **Table 3**.

**Table 3:** Accuracy comparison experiments for different backbone networks.

Method	VGG16	ResNet50	mIoU	mPA
UNet-1		√	85.34%	91.27%
UNet-2	√		86.69%	92.52%
UNet-3	√		87.50%	93.36%

The training loss curves and validation loss curves for the two backbone networks are displayed in **Supplementary Figures 11A, B**, respectively. The 100 training rounds’ worth of loss curves are shown in **Supplementary Figure 11A**. **Supplementary Figure 11A** shows that the loss values of both backbone networks on the training set first decrease fast and then roughly level out, indicating better convergence. On the other hand, the validation loss curve offers a better representation of the network’s performance on brand-new, untested data.

The validation loss values for ResNet50 swing noticeably in the later phases of training, as seen by the loss curves for the validation set in **Supplementary Figure 11B**. This suggests that the network’s ResNet50 structure has been severely overfitted. The validation loss values of VGG16, however, stay smooth and essentially converge in the late training period, demonstrating that the model has attained a respectable training effect.

### 3.4 Ablation experiments

In order to verify the improvement of segmentation performance of improved UNet, this paper conducts segmentation performance ablation experiments on the self-made optical Betula luminifera data set. Replace the backbone feature extraction network of UNet itself with VGG16, add the ASPP module between the backbone feature extraction module and the enhanced feature extraction module, add the CCA attention module to each feature layer of the enhanced feature extraction module, and introduce Dice_Boundary Loss, setting Nine model experimental comparisons are detailed as follows:

UNet-D: In the traditional UNet model, the Dice_Boundary Loss loss function is introduced;

UNet-VD: Based on UNet-D, the backbone feature extraction network is replaced by VGG16;

UNet-AD: Based on UNet-D, the ASPP module is added between the backbone feature extraction module and the enhanced feature extraction module;

UNet-CD: Based on UNet-D, the CCA attention module is added after strengthening each feature layer of the feature extraction module;

UNet-VAD1: Based on UNet-VD, the ASPP (1,6,12,18) module is added between the backbone feature extraction module and the enhanced feature extraction module;

UNet-VAD2: Based on UNet-VD, the ASPP(1,2,4,8) module is added between the backbone feature extraction module and the enhanced feature extraction module;

UNet-VAD3: Based on UNet-VD, the ASPP(1,2,7,15) module is added between the backbone feature extraction module and the enhanced feature extraction module;

UNet-VCD: Based on UNet-VD, the CCA attention module is added after strengthening each feature layer of the feature extraction module;

UNet-VACD: Based on UNet-VAD, the CCA attention module is added after strengthening each feature layer of the feature extraction module, which is the method in this article.

**Table 4** details the performance gains obtained through the combination of different modules. The input size of the training images is 512*512. From the results of the ablation experiment, it can be seen that comparing UNet and UNet-D, the mIoU, mPresicion, and mPA indicators have improved. It shows that introducing the Dice_Boundary Loss loss function into the model has certain benefits in improving the accuracy of the model.

**Table 4:** Results of ablation experiments.

Method	mIoU	Presicion	mPA
UNet	84.45%	92.02%	91.11%
UNet-D	86.03%	92.10%	91.73%
UNet-VD	86.69%	92.49%	92.52%
UNet-AD	86.71%	92.32%	92.48%
UNet-CD	86.68%	92.30%	92.45%
UNet-VAD1	86.95%	92.79%	92.54%
UNet-VAD2	87.08%	92.79%	92.71%
UNet-VAD3	87.16%	92.75%	92.84%
UNet-VCD	86.80%	92.60%	92.53%
UNet-VACD	87.50%	93.69%	93.36%

Comparing UNet and UNet-VD, the index values of UNet-VD in mIoU, mPresicion, and mPA have all increased, confirming that replacing the feature extraction network with VGG16 is the main factor in improving the model segmentation performance.

Comparing the three models UNet-VAD1, UNet-VAD2, and UNet-VAD3, the indicators in mIoU, mPresicion, and mPA are all higher than UNet-D. The index of UNet-VAD3 is the best among the three, that is, the segmentation performance of this model is better, indicating that the dilation rate of (1, 2, 7, 15) used in dilated convolution is the main factor in improving segmentation performance.

Compared with UNet-D, UNet-VD, UNet-AD, and UNet-CD, the mIoU, mPresicion, and mPA indicators have all improved. It shows that both dilated convolution ASPP and cross-attention mechanism CCA can improve the segmentation performance of the model to a certain extent. Among them, UNet-AD has a higher degree of improvement in performance indicators than the other three models. Therefore, the addition of ASPP is an overall improvement. The workhorse of model segmentation performance.

Comparing UNet-VCD and this article’s method UNet-VACD, the replacement of the backbone feature network and the addition of CCA did not significantly improve the efficiency. The introduction of ASPP improved the segmentation accuracy of the model to a certain extent. This comprehensive evaluation process allows us to evaluate the performance and stability of the proposed AC-UNet model more efficiently.

### 3.5 Performance comparison of different segmentation models

The method presented in this paper is compared with several advanced segmentation methods, namely PSPNet, DeepLabV3, UNet and Swin-UNet, on the Betula luminifera dataset. To ensure the rigor and fairness of the comparison experiments, all segmentation methods employ the same experimental equipment, uniform image size, identical parameter set-tings, and consistent training processes. The differences in data, including mIoU, mPA, FLOPs, and Params, between the methods are evaluated and compared. As depicted in **Table 5**, the AC-UNet plant stem and leaf segmentation model, designed in this paper, exhibited the best segmentation performance.

**Table 5:** Comparison of evaluation indicators for different segmentation models.

Method	mIoU	mIoU_stem	mPA	Precision	F1	FLOPs/G	Params/M
		mIoU_leaf					
PSPNet	58.76%	26%	73.24%	66.90%	69.93%	6.031	2.376
		52%					
DeepLabV3	82.13%	64%	91.47%	87.73%	89.56%	52.875	5.814
		83%					
UNet	84.45%	70%	91.11%	90.63%	92.38%	451.706	24.891
		85%					
Swin-UNet	79.02%	57%	85.99%	88.73%	87.36%	91.497	41.342
		80%					
AC-UNet	87.50%	75%	93.36%	93.69%	93.52%	482.001	34.244
		88%					

**Table 5** show the results of comparing AC-UNet with the remaining four networks. The mIoU, mPA, Precision, and F1, all four metrics of the method model in this paper are higher than the remaining three networks, with F1 = 93.52%, indicating the excellent learning performance of the model. More importantly, AC-UNet performed best in the metric of mIoU, achieving considerable improvement over the other network models. It is evident from **Supplementary Figure 12** that Ours outperforms PSPNet, DeepLabV3 and UNet by around 28.32%, 4.95%,3.05% and8.48%, respectively, with segmentation values for stem improving by 49%, 11%, 5% and 18%, respectively, and segmentation values for leaf improving by 36%, 5%, 3% and 8%, respectively.

The DeepLabV3 and UNet classical networks’ respective parameters for the network are 2.376M, 5.184M, and 24.891M, respectively, with the exception of PSPNet, as shown by the four different models. The difference in mPA was not perceived to be very significant, despite the fact that the number of parameters changed significantly, particularly for DeepLabV3 and classical UNet, which have respective mPAs of 91.47% and 91.11%. In addition, Swin-UNet has the smallest model complexity and can segment stems and leaves in a short time. However, in addition to its speed advantage, it fails to provide better segmentation results. It is evident from **Table 5** that DeepLabV3 and UNet have a lot more parameters that the network used in this study—5.814 and 24.891, respectively.

This indicates that the segmentation effect is not necessarily enhanced due to network lightweighting in the case of a small number of parameters. The mIoU, mPA and Precision, of the equally lightweight PSPNet are only 58.76%, 73.24% and 69.93%, respectively, which are less effective in segmentation. It can be seen that the lightweighting changes are not necessarily applicable to the needs of the segmentation algorithm in this paper.

The relationship between segmentation losses and the total number of iterations was discovered using the earlier suggested technique for segmentation training on Betula luminifera data, as shown in **Supplementary Figure 13A, B**. It can be clearly seen from **Supplementary Figure 13A** that AC-UNet has a lower loss value in the initial stage. The algorithm has excellent phenotypic fitting ability and better generalization ability. As can be seen from **Supplementary Figure 13B**. The segmentation loss becomes lower and smaller as the number of iterations rises. For PSPNet, the loss suddenly decreases after 50 iterations and then tends to level off. At around 60 repetitions, the segmentation loss for the remaining three networks starts to stabilise. It is essential to note that the network utilised in this paper’s technique has a tiny initial loss value, showing that its convergence impact is far better than that of the other networks. This shows that the network presented in this paper’s convergence impact has a good convergence effect.

**Supplementary Figure 14** is an example of model segmentation output, where A is the original image, B is the label, and the rest C, D, and E are the fusion prediction map of the label and the original image, the stem prediction map and the leaf prediction map. This example segmented output image shows images of three different morphologies of Betula luminifera, including leaves and stems. The image is segmented into regions where stems are marked in green and leaves in red. However, there are faults or fuzzy boundaries in some places, such as the junction of stems and leaves, and the edges of leaves. The model may not be able to clearly distinguish them due to the occlusion of the stems by the leaves of the plants. Overall, the algorithm in this paper performs well on the example image, and most of the stems and leaves are accurately segmented. However, it should also be noted that for some complex areas, further optimization is still required.

The segmentation has been portrayed using a custom dataset in order to demonstrate how the performance of the method in this research differs from that of other methods. The dataset’s visualisation results are shown in **Supplementary Figure 15**, where (A) depicts the original image, (B) the label, and the images that remain (C), (D), (E), (F) and (G), respectively, correspond to PSPNet, DeepLabV3, UNet, Swin-UNet, and the approach suggested in this paper, AC-UNet.

**Supplementary Figure 15C**-PSPNet shows a substantial inaccuracy in segmentation accuracy. The image is barely segmented out in the C column, with only vaguely distinguishable stems and leaves. It has no reference point compared to the rest of the methods. In **Supplementary Figure 15D**-DeepLabV3, the segmentation effect is rough, and the branch and stem parts appear disconnected. A comparison reveals that the segmentation treatment of the detail part in this paper’s method is closer to the label, providing higher segmentation accuracy. **Supplementary Figure 15E**-UNet and **Supplementary Figure 15G**-AC-UNet both achieve better segmentation results, but ours demonstrates more pragmatic segmentation results for mutilated leaves and better detailing of the edges of leaves and stems. **Supplementary Figure 15F** -Swin-UNet can be clearly seen with the naked eye, and some stem and leaf segmentations appear to be mis-segmented, segmented and stacked. In contrast, AC-UNet shows better results in stem and leaf details, incomplete leaf segmentation, and leaf edge segmentation. Therefore, the performance of the algorithm in this paper is more stable and less prone to the above problems, which makes it a more reliable algorithm choice. It can be seen from the segmentation effect that AC-UNet has a higher-precision segmentation benefit in the stem and leaf segmentation task. This benefit has obvious advantages in the later measurement of phenotype correlation coefficients, ensuring that it can be used in specific environments. Stability and reliability.

Methods of this paper demonstrates better and superior segmentation results for plant stems and leaves.

### 3.6 Phenotypic analysis of Betula luminifera

Select any 15 groups of plant objects from the Betula glabra dataset and evaluate and predict the crown area of the plants. The calculation of the crown area in this paper is evaluated by referencing half of the product of the plant height and crown width. The segmentation network proposed in this paper is used for image prediction, and the predicted image is obtained as shown in **Supplementary Figure 16A**. Using HSV color threshold segmentation, select the red threshold part in the mask image for segmentation and extraction. Refer to **Table 1** in Section 2.3 for the values. The segmentation effect is shown in **Supplementary Figure 16C**. Finally, through OTSU, the leaf part we need, which is the foreground part in **Supplementary Figure 16C**, is segmented and extracted. Lastly, the corresponding feature grayscale binary image is obtained, as shown in **Supplementary Figure 16D**. Based on the binary image, the crown area of the plant is calculated.

Take three groups of plants with different shapes from the visual analysis for binarization, and analyze the proportion of stems and leaves in the image (because the background occupies a large area, we take 10 as the whole image). At the same time, due to the segmentation effect of Swin-UNet There is a large error and overlap, so Swin-UNet is not included in the comparison. It can be seen from the stacked **Supplementary Figure 17** (where A, B, C, and D respectively represent PSPNet, DeepLabV3, UNet, and the method AC-UNet in this article, and 1, 2 and 3 are label maps) that in each subgroup of A, B, and C, except for B3 They are all significantly different from the proportion of stems and leaves in the label map. The distribution of the proportion of stems and leaves in each group of category D is approximately the same as that in the label map.

We determined the proportionate relationship between the image and the actual plant based on the height and length of the crown cross-section of the actual, measured plant. The actual values and the expected results were then compared and analysed. The data’s regression analysis reveals that the model fits the data well because the R2 value for leaf is 0.99882, which is greater than 0.8 and near to 1. The 15 data points are all close to the regression line in **Supplementary Figure 18**, illustrating a high positive correlation between the predicted and actual values.

This calculation method eliminates the need for destructive sampling of plants and facilitates the development of continuous dynamic observation of the same research object while reducing the time required for determination in terms of manpower on a large number of samples compared to conventional methods. These methods include the squared paper method, the paper-cut weighing method, the leaf-area-meter determination method, and the image-processing method through scanning and photographing. The design incorporation of CCA and ASPP in the network expands the contextual horizon in the deep learning process, allowing the network to learn more detailed information during the training process, thereby facilitating better plant segmentation performance. Further-more, it is more evident from the segmentation results that the fusion of attention mechanism and ASPP in UNet leads to further enhancement in refining plant image details. This enhancement provides strong support for the final realization of plant phenotypic traits acquisition

## 4 Discussion

Earlier vision-based segmentation research was more applied in remote sensing images (urban, agriculture and forestry) and crops. Hong and his collaborators (Hong et al., 2021), in view of the limitations of convolutional neural networks (CNNs) when sampling, incorporated an improved batch GCN (miniGCN) and proposed an end-to-end network FuNet with a fusion strategy to ensure network stability On the basis of reducing computational costs, high-efficiency remote sensing image segmentation is achieved, which opens up new ideas for solving restrictive problems in the segmentation field. In addition, Hong et al. (Hong et al., 2023)also introduced HighDAN, a high-resolution domain-adaptive network architecture that can solve cross-city or region problems. The network achieved the best segmentation performance on the constructed multi-modal remote sensing benchmark data set (C2Seg data set). Solve bottlenecks that hinder urban planning and development. Some of them have made corresponding improvements to the UNet model and achieved good results (Genze et al., 2023; Wang et al., 2023). However, some advanced segmentation models based on deep learning are not completely suitable for plant stem and leaf segmentation. This is because plant stems and leaves contain more feature information than the plant as a whole, but occupy a smaller proportion of pixels in the image, making segmentation difficult.

In this study, we demonstrated the advantages of AC-UNet in stem-leaf segmentation in Betula luminifera populations and the convenience it brings in the acquisition of plant phenotypic traits in the later stage, which is expected to be used in the field of tree species segmentation and plant phenotypic traits acquisition make a certain contribution. This is of great significance for early plant breeding and species health assessment. The mIoU value of AC-UNet in the segmentation of plant stems and leaves reached 87.5%, confirming that the method overcomes the effect of extracting stem and leaf detail information to a certain extent due to problems such as missing edge information, faults at the junction of stems and leaves, and uneven image samples. Almost difficult. The proposed method still faces some limitations: increasing the data of Betula luminifera in other different geographical areas, enhancing the rationality and universality of the research; AC-UNet performed well in extracting details of, conifers, shrubs, etc.) needs to be further explored; study the lightweight of the network, and improve its efficiency on the basis of ensuring the segmentation accuracy of the network model; solve the problem of leaf occlusion during the growth of plants, in order to further improve the performance of the network model. Acquisition accuracy of type traits.

## 5 Conclusion and outlook

The existing semantic segmentation network cannot achieve better segmentation of plant stems and leaves. The extraction of plant edges, joints, and details is poor, and accurate segmentation of organs such as stems and leaves is not possible. In order to solve these problems, this paper proposes a new segmentation network called AC-UNet, which addresses the segmentation challenges specific to Betula luminifera’s stem and leaf organs. Considering the unique characteristics of leaf edge details and stem-leaf connections in the segmentation prediction process of Betula luminifera’s stem and leaf organs, the AC-UNet algorithm, an improved version of UNet, is introduced. This algorithm aims to enhance the overall segmentation accuracy by addressing the issues of insufficient edge information and disconnections in conventional segmentation algorithms. Additionally, a composite loss function called Dice_Boundary, which combines the Dice and Boundary metrics, is introduced at the back-end of the network to tackle the problem of imbalanced image samples.

This paper focuses on experimental observations using Betula luminifera seedlings planted in the northwest of Zhejiang Province. A performance comparison is conducted among different models including PSPNet, DeepLabV3, and UNet, and based on the results, an improved AC-UNet model is designed on the foundation of UNet. The experimental results demonstrate that AC-UNet significantly enhances the accuracy of stem and leaf segmentation for Betula luminifera, achieving an mIoU value of 87.50% and accurately extracting detailed parts of the plant. Follow-up research will focus on planting Betula luminifera seedlings and other tree species (broad leaves, conifers, shrubs, etc.) in different geographical locations to support the universal applicability of this algorithm in obtaining plant phenotypic information. Future applications will expand from stem and leaf segmentation to tree segmentation, botany, etc., and provide new core technology research paths.

## Data availability statement

The raw data supporting the conclusions of this article will be made available by the authors, without undue reservation.

## Author contributions

XY: Supervision, Writing – review & editing. JW: Software, Writing – original draft. PW: Conceptualization, Methodology, Writing – review & editing. GW: Formal Analysis, Writing – review & editing. LM: Resources, Writing – review & editing. XL: Data curation, Writing – review & editing. HL: Visualization, Writing – review & editing. HH: Funding acquisition, Writing – review & editing. EL: Data curation, Writing – review & editing. BM: Investigation, Writing – review & editing. CL: Writing – review & editing.
