# Supplementary figures and images for: AC-UNet: an improved UNet-based method for stem and leaf segmentation in Betula luminifera
Source: Front Plant Sci. 2023 Nov 27;14:1268098. doi: 10.3389/fpls.2023.1268098 (PMC10711104; doi:10.3389/fpls.2023.1268098)

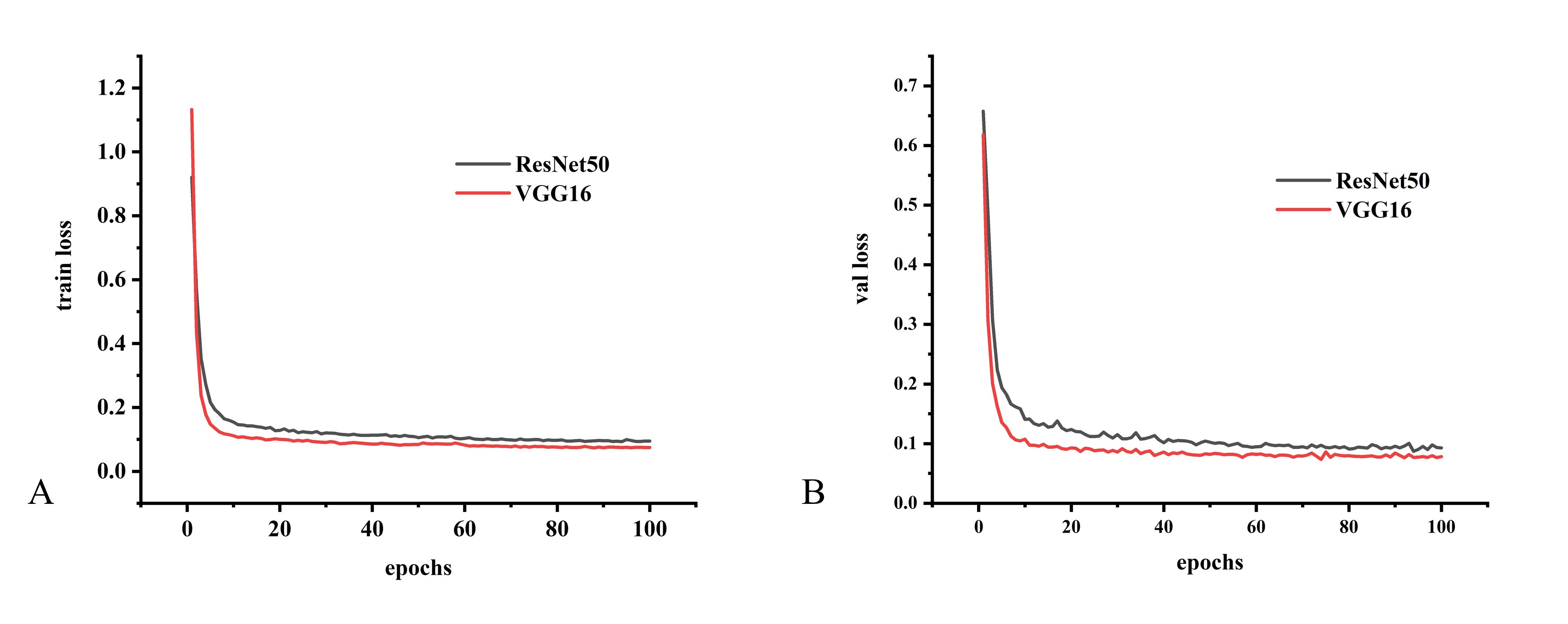

Supplement: Supplementary file 1 [file Image_1.jpeg]

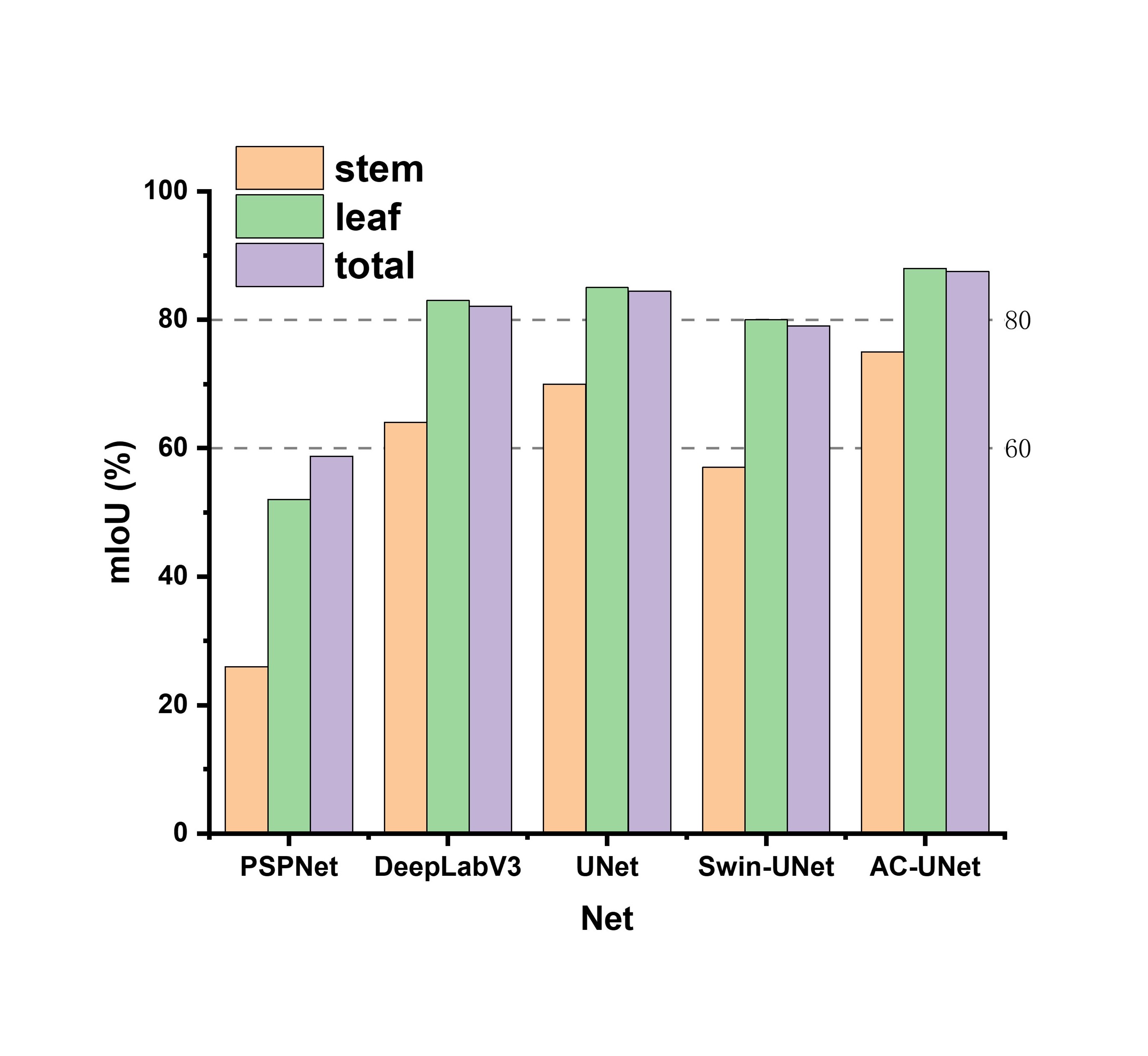

Supplement: Supplementary file 2 [file Image_2.jpeg]

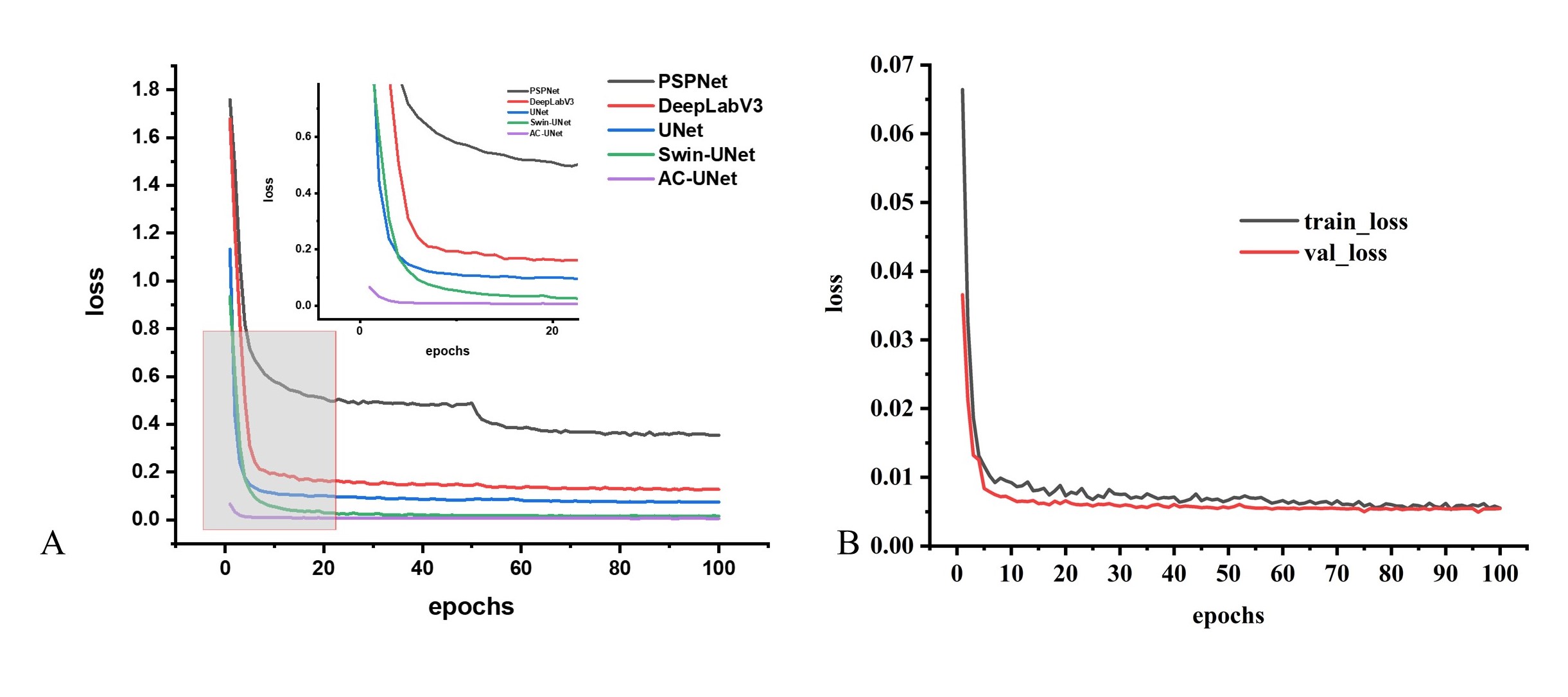

Supplement: Supplementary file 3 [file Image_3.jpeg]

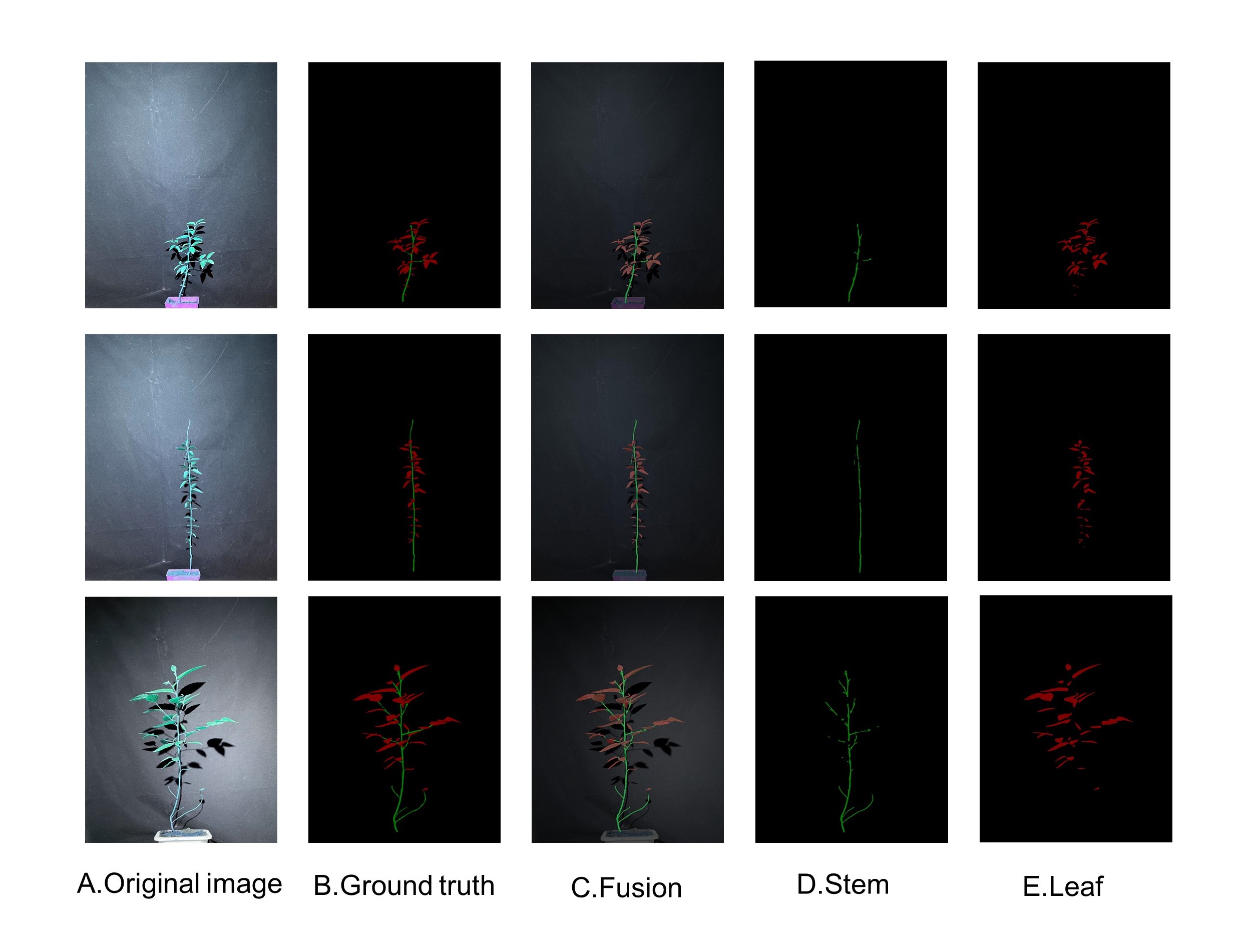

Supplement: Supplementary file 4 [file Image_4.jpeg]

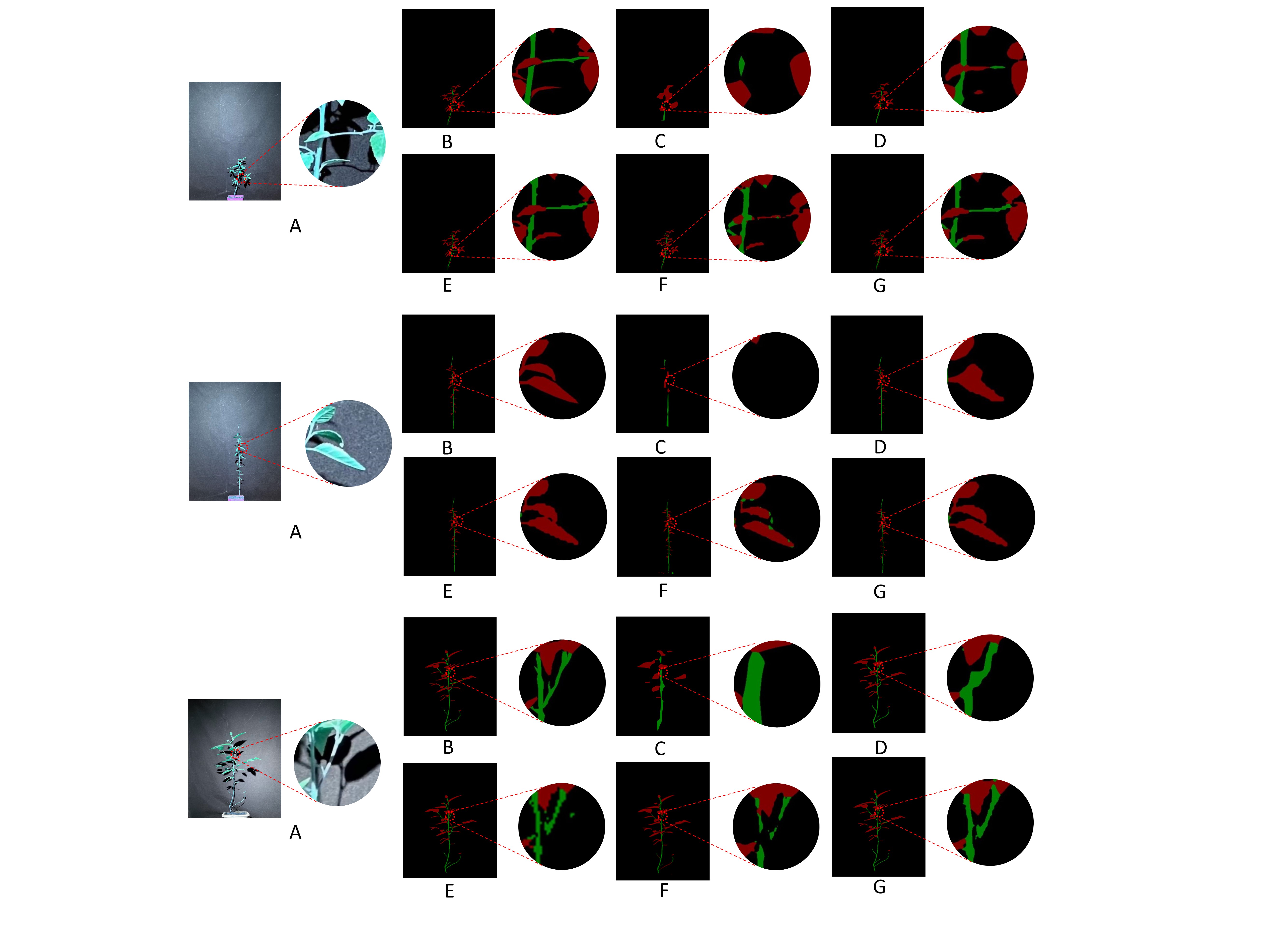

Supplement: Supplementary file 5 [file Image_5.jpeg]

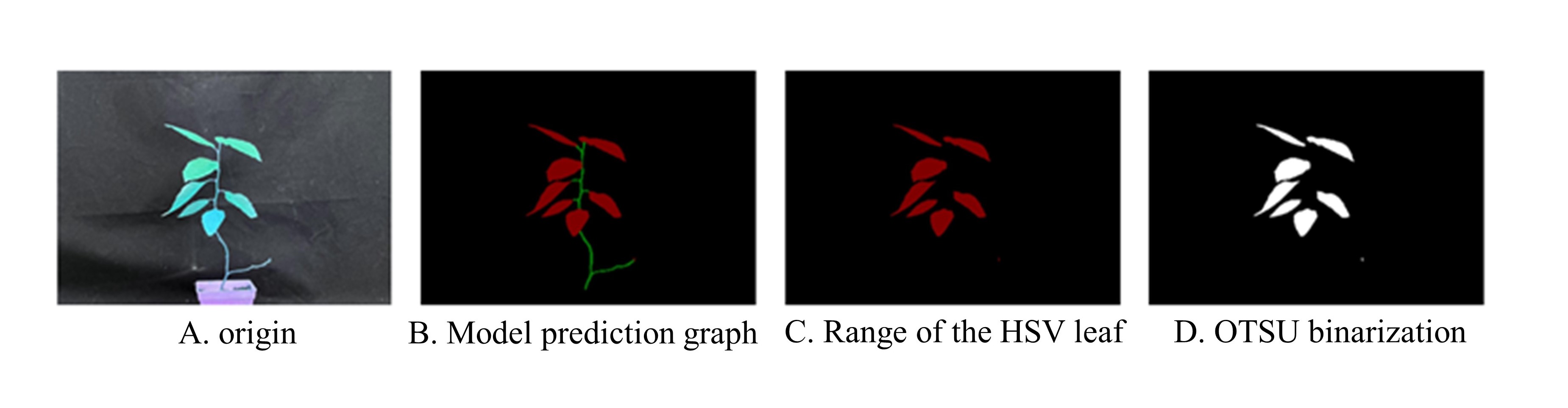

Supplement: Supplementary file 6 [file Image_6.jpeg]

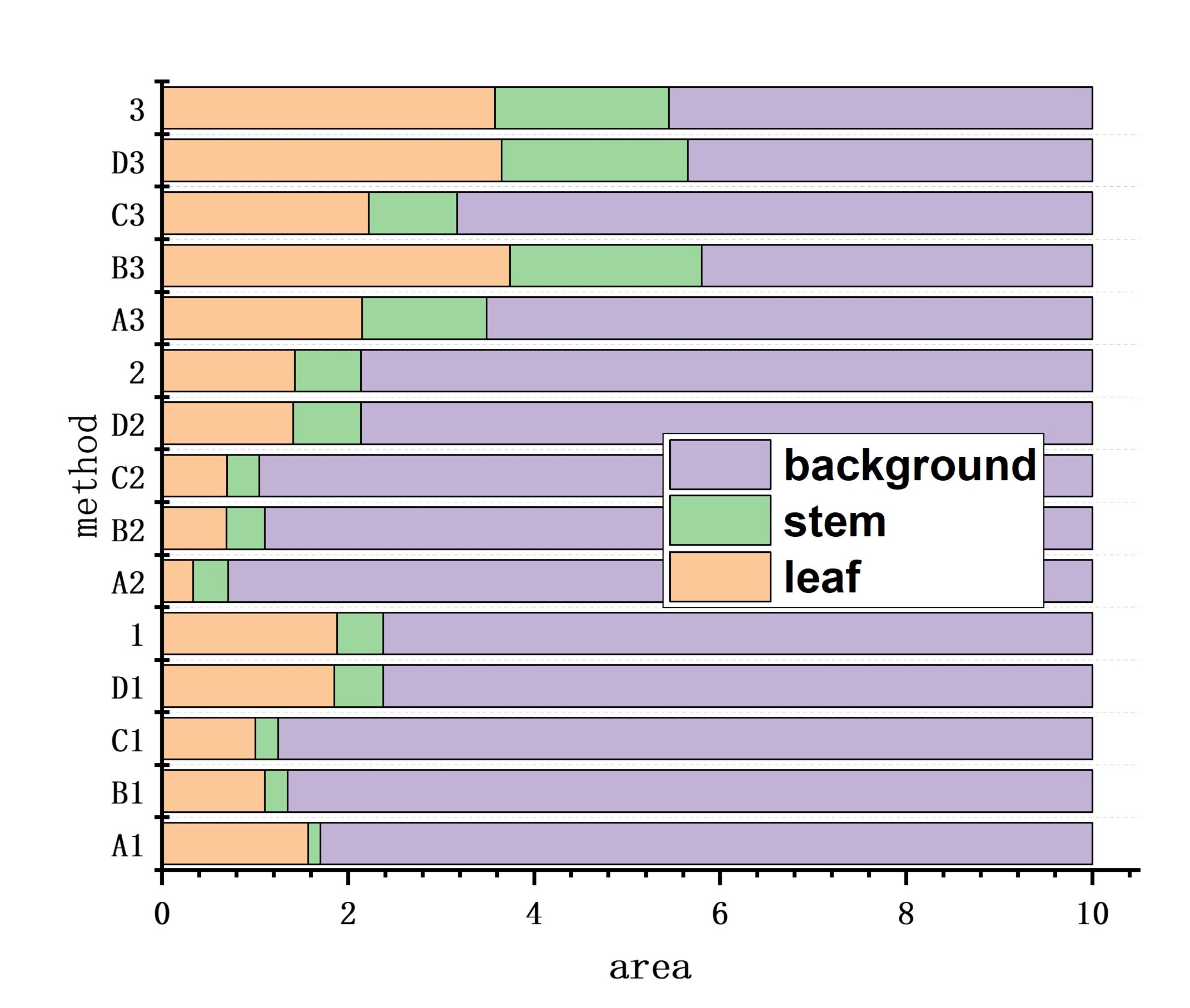

Supplement: Supplementary file 7 [file Image_7.jpeg]

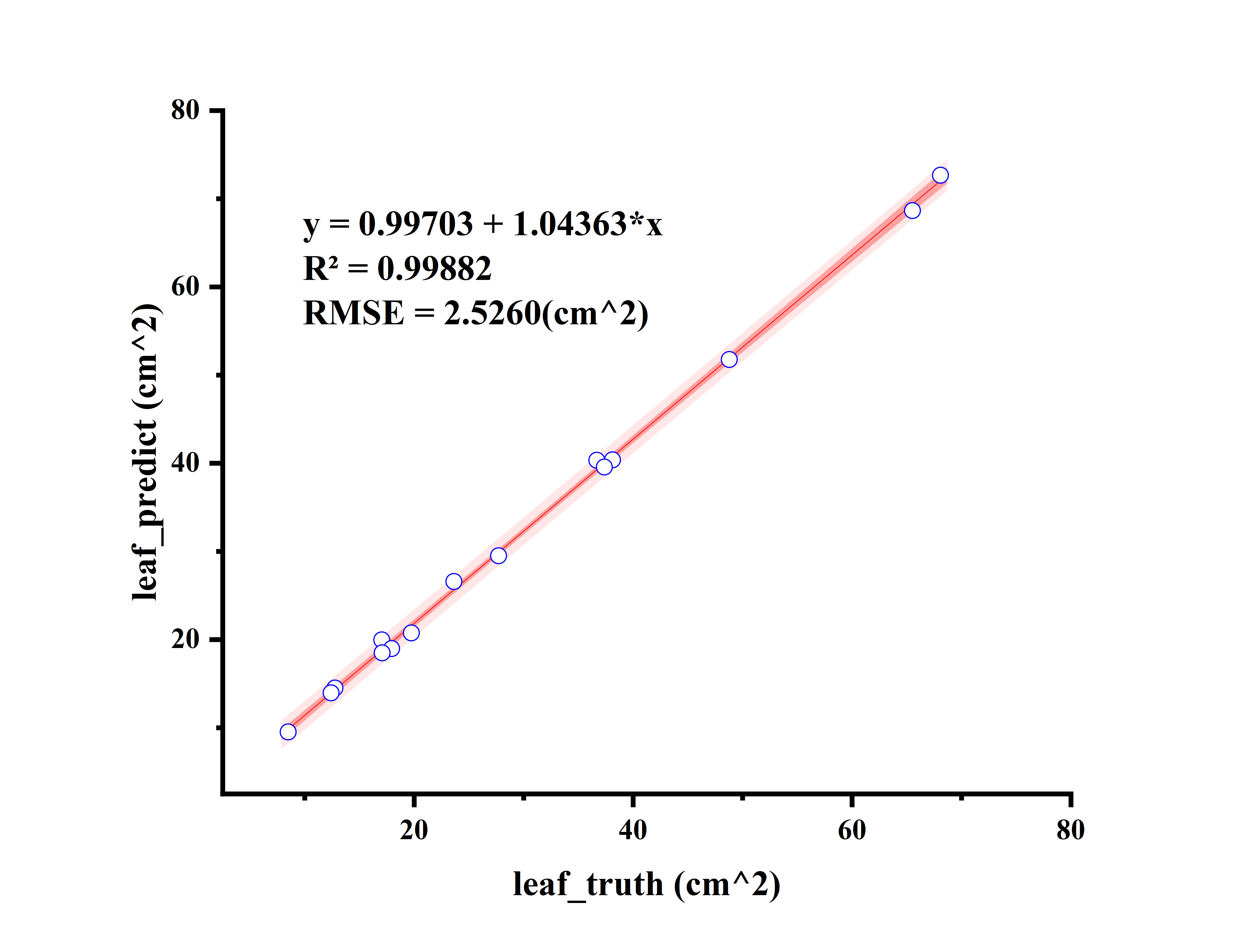

Supplement: Supplementary file 8 [file Image_8.jpeg]
